# Production of Indole-3-Lactic Acid by *Bifidobacterium* Strains Isolated fromHuman Infants

**DOI:** 10.3390/microorganisms7090340

**Published:** 2019-09-11

**Authors:** Takuma Sakurai, Toshitaka Odamaki, Jin-zhong Xiao

**Affiliations:** Next Generation Science Institute, Morinaga Milk Industry Co., Ltd., Zama, Kanagawa 252-8583, Japan; t_sakura@morinagamilk.co.jp (T.S.); t-odamak@morinagamilk.co.jp (T.O.)

**Keywords:** *Bifidobacterium*, indole-3-lactic acid, indole-3-propionic acid, indole-3-acetic acid, indole-3-aldehyde

## Abstract

Recent studies have shown that metabolites produced by microbes can be considered as mediators of host-microbial interactions. In this study, we examined the production of tryptophan metabolites by Bifidobacterium strains found in the gastrointestinal tracts of humans and other animals. Indole-3-lactic acid (ILA) was the only tryptophan metabolite produced in bifidobacteria culture supernatants. No others, including indole-3-propionic acid, indole-3-acetic acid, and indole-3-aldehyde, were produced. Strains of bifidobacterial species commonly isolated from the intestines of human infants, such as *Bifidobacterium longum* subsp. *longum*, *Bifidobacterium longum* subsp. *infantis*, *Bifidobacterium breve*, and *Bifidobacterium bifidum*, produced higher levels of ILA than did strains of other species. These results imply that infant-type bifidobacteria might play a specific role in host–microbial cross-talk by producing ILA in human infants.

## 1. Introduction

Tryptophan can be metabolized by microbiota organisms. Tryptophan metabolites, including indole-3-lactic acid (ILA), indole-3-propionic acid (IPA), indole-3-acetic acid (IAA), and indole-3-aldehyde (IAld), play important roles in host homeostasis. These tryptophan metabolites have been reported to act as agonists of the aryl hydrocarbon receptor and farnesoid X receptor (FXR) [1,2]. IAA can suppress inflammatory responses of cytokine-mediated lipogenesis in hepatocytes via the reduction of pro-inflammatory cytokine production in macrophages [3]. IAld stimulates lamina propria lymphocytes to secret IL-22 and restores the barrier function of damaged intestinal mucosa by, in turn, stimulating the proliferation of intestinal epithelial cells [4]. IPA regulates gastrointestinal barrier functions by the downregulation of enterocyte tumor necrosis factor-α (TNF-α) and the upregulation of junctional proteins [5]. It has also been reported that these tryptophan metabolites can inhibit amyloid fibrillation of lysozymes and that they possess neuroprotective properties [6]. ILA scavenges free radical products and inhibits the UVB-induced production of interleukin-6 (IL-6) [7,8]. ILA was reported to reduce TH17 polarization which suppresses inflammatory T cells and gut intraepithelial CD4^+^CD8αα^+^ T cells (immunoregulatory T cells) [9,10]. ILA acts as an agonist of human hydroxycarboxylic acid receptor 3 and induces a decrease in cAMP in human monocytes [11]. It has been reported that Bifidobacterium strains produce ILA [12]. However, to the best of our knowledge, information relating to tryptophan metabolite-producing *Bifidobacterium* strains is scarce [13].

*Bifidobacterium* strains commonly found to colonize the human gut are designated as human-residential bifidobacteria (HRB), while Bifidobacterium strains that naturally colonize the gut of other animals are referred to as non-HRB. *B. breve*, *B. longum* subsp. *infantis*, *B. bifidum*, and *B. longum* subsp. *longum* are most frequently observed *Bifidobacterium* species in human infants (infant-type HRB) [14,15,16,17,18]. It is important to note that the distribution of bifidobacterial species changes with host age, which is caused by age-related changes in dietary habits [19]. Dominant HRB in adults are referred to as adult-type HRB.

The present study aimed to evaluate the capacity of *Bifidobacterium* strains to produce various tryptophan metabolites (ILA, IAA, IAld, and IPA). We first examined 19 typical strains that are available from public culture collection facilities. Then, the ability of 100 newly isolated strains [20] to produce ILA was examined.

## 2. Results and Discussion

### 2.1. Production of Tryptophan Metabolites by Bifidobacterium strains

To begin with, 19 bifidobacterial strains obtained from culture collections were tested by culturing in de Man, Rogosa and Sharpe (MRS) (Table 1). No obvious differences in growth were observed. MRS did not contain ILA (< 0.005 µg/mL), and explicit production of ILA was observed in culture supernatants (CSs). The average concentration of ILA in CSs of infant-type HRB (*B. longum* subsp. *longum*, *B. longum* subsp. *infantis*, *B. breve*, and *B. bifidum*) was higher compared with other strains (Table 1). Other tryptophan metabolites (IAld, IAA, and IPA) were not produced by any of the strains tested (Figure S1). To confirm the differences in ILA production among each of the *Bifidobacterium* species, a total of 100 newly isolated strains were also tested. Figure S2 shows the concentration of ILA in the CSs of these 100 strains, and the data are summarized in Table 2. The average concentration of ILA in CSs of *B. longum*, *B. breve*, *B. bifidum*, and *Bifidobacterium kashiwanohense* was higher than in CSs of *Bifidobacterium pseudocatenulatum*, *Bifidobacterium adolescentis*, and *Bifidobacterium dentium*.

### 2.2. Discussion

Microbiota-derived tryptophan metabolites play important roles in their hosts’ homeostasis [21,22]. Some bifidobacterial strains produce ILA, IAA, and IPA [13]. In this investigation, we tested the ability of various bifidobacterial strains to produce tryptophan metabolites (IAld, IAA, IPA, and ILA). We observed only the production of ILA by bifidobacterial strains (Table 1). These tryptophan metabolites are found in plants as auxins or their intermediates, and MRS broth containing a digest of soybean, we suppose that IAld, IAA, and IPA were derived from the ingredients of MRS broth [23,24]. The same results were observed not only in MRS broth CSs but also in Gifu Anaerobic Medium (GAM) broth CSs (Table S2). In addition, we found that the ability to produce ILA reflected strain-specific features. That is *B. longum* subsp. *longum*, *B. longum* subsp. *infantis*, *B. breve*, and *B. bifidum*, which are usually found in the intestines of human infants and designated infant HRBs [16,25], produced relatively higher levels of ILA compared with the other strains (Table 1). We further investigated the production of ILA by 100 newly isolated bifidobacterial strains [20]. The production of ILA by infant HRBs was significantly higher than the production of this compound by *B. pseudocatenulatum*, *B. adolescentis*, and *B. dentium*. We did not examine the type-strain of *B. kashiwanohense*, which has previously been isolated from the feces of healthy infants [26]. Therefore, although we recognize that *B. kashiwanohense* can be classified as an infant HRB, there were too few *B. kashiwanohense* CSs to judge the results.

The mechanism of the production of ILA from bifidobacterial strains was not clarified in this study. However, we suppose two metabolic pathways for the ILA production by infant-type HRB. One possible pathway is through tryptophan deamination by amino acid oxidase (AAO) [27]. Another metabolic pathway is a conversion from tryptophan to indolepyruvic acid by aromatic amino acid aminotransferase (Aat), followed by conversion to ILA by phenyllactate dehydrogenase (fldH) [28], although the related gene was not identified in this study. 

Our result suggests that further investigation of ILA biological meaning is needed to fully understand how and why only limited species (infant-type HRB) are allowed to harbor in the human infant gut. As described in the introduction, ILA has been reportedly involved in inducing immunoregulatory T cells [9,10] and suppressing inflammatory T cells [29,30,31,32]. This would be one of the benefits for normal growth, including the immune development in infants. From the bacteria aspect, we speculate that ILA production by infant-type HRB may contribute to the predominance of themselves in the infant’s large intestine because ILA was reported to have antimicrobial activity [33] in addition to H_2_O_2_ production as a by-product during tryptophan deamination [34].

## 3. Materials and Methods

### 3.1. Materials

Indole-3-lactic acid (ILA) was purchased from Tokyo Chemical Industry Co., Ltd. (Chuo-ku, Tokyo, Japan). Indole-3-propionic acid (IPA), indole-3-acetic acid (IAA), indole-3-carboxaldehyde (IAld), and 3-methyl-2-oxindol (MOI) were purchased from Merck, Japan (Tokyo, Japan). Acetonitrile (HPLC grade) was purchased from Kanto Chemical Co., Ltd. (Tokyo, Japan). Ammonium acetate (AA) was purchased from Merck, Japan. Unless otherwise stated, all chemical reagents used were of analytical grade.

### 3.2. Bacterial Strains

Bifidobacterial strains were obtained from the Morinaga Culture Collection (Morinaga Milk Industry Co., Ltd., Zama, Japan) or purchased from the American Type Culture Collection (Manassas, VA, USA), the Japan Collection of Microorganisms (Wako, Japan), the German Collection of Microorganisms (DSMZ; Braunschweig, Germany), or the Laboratorium voor Microbiologie (LMG; Belgium). A further 100 newly isolated strains, which were reported in a previous study [25], were also used.

All strains were individually cultured under anaerobic conditions in MRS broth (Becton Dickinson, MD, USA) supplemented with 0.05% L-cysteine (Kanto Chemical Co., Ltd., Chuo-ku, Tokyo, Japan)) (MRS-C) using an Anaero Pack (Mitsubishi Gas Chemical, Tokyo, Japan).

### 3.3. Culture Supernatants (CSs)

Initially, all bifidobacterial strains tested were maintained by culturing at 37 °C for 16 h under anaerobic conditions in MRS-C. The growth-phase bacterial cells were then harvested by centrifugation [high-speed centrifugal refrigerating machine, HIMAC SCR20B (Hitachi Koki Co., Ltd., Tokyo, Japan)] at 5000× *g* (4 °C for 10 min) and washed twice with phosphate buffered saline (PBS) and Dulbecco’s Formula (DS Pharma Biomedical Co., Ltd., Osaka, Japan). Subsequently, whole-cell pellets were suspended in PBS containing 0.05% L-cysteine (PBS-C). The optical density (at 600 nm) of each bacterial cell suspension was adjusted to the same value (OD600 = 0.2) using PBS-C. Cell suspensions (100 µL) were added to MRS-C (3 mL) and cultured at 37 °C for 24 h under anaerobic conditions. The CSs were obtained by centrifuging the culture suspensions at 5000× *g* (4 °C for 10 min). Following filtration (pore size 0.22 µm; Millipore, MA, USA), the samples were stored at −80 °C until use. All cultures were grown in independent triplicates, and the resulting data were expressed as the mean of these replicates.

### 3.4. Quantification of Tryptophan Metabolite Concentrations in CSs

The concentration of the four metabolites in CSs was analyzed using liquid chromatography–tandem mass spectrometry (LC-MS/MS; TSQ Quantum Discovery Max, Thermo Electron Corp., San Jose, CA, USA). Chromatographic separation was performed using an InertSustain C18 column (GL Science Inc., Tokyo, Japan) (2.1 × 150 mm, 2 µm). Mobile phase A (containing 1 g/L AA in water) and mobile phase B (containing 1 g/L AA in acetonitrile) were applied at a flow rate of 0.2 mL/min. The gradient elution was started at 10% B. At 0.1–18 min, 10%–90% B; 18.1–25 min, 90%; 25.1–28 min, 90%–10%; 28–40 min, 10%.

Quantitation was performed by comparing metabolite concentrations in CSs with those of the corresponding synthetic compound standards (IAA, IAld, IPA, and ILA) and the internal standard (MOI). The LC–MS/MS spectrum (product ion data) of the positive precursor ion was evaluated to determine their final content (Table S1).

### 3.5. Statistical Analyses

Intergroup differences in ILA production were analyzed using unpaired *t*-tests. *p* values < 0.001 were considered statistically significant.

## 4. Conclusions

In conclusion, we examined the ability of various bifidobacterial strains to form tryptophan metabolites. We found that typical infant-type HRB produced significantly higher concentrations of ILA compared with adult-type HRB and non-HRB. Future investigations of ILA-producing microbiota will help to further reveal the role of infant-type HRB in the human gut.

## Figures and Tables

**Table 1:** Production of Indole-3-Lactic Acid (ILA) by 19 Bifidobacterial Strains.

Species	Isolated from	Strain	ILA (µg/mL)	OD600
*B. bifidum*	Infant feces	ATCC 29521^T^	4.9 ± 0.4	0.7 ± 0.1
	Infant feces	NITE BP-02429	3.4 ± 0.5	0.7 ± 0.0
	Infant feces	NITE BP-02431	2.4 ± 0.1	0.7 ± 0.0
*B. breve*	Intestine of infant	ATCC 15700^T^	2.0 ± 0.2	1.0 ± 0.1
	Infant feces	FERM BP-11175	2.6 ± 0.3	1.0 ± 0.0
	Infant feces	NITE BP-02622 (M-16V)	4.4 ± 0.5	1.0 ± 0.1
*B. longum* subsp. *infantis*	Intestine of infant	ATCC 15697^T^	3.3 ± 0.5	1.1 ± 0.0
	Intestine of infant	NITE BP-02623 (M-63)	3.1 ± 0.3	1.3 ± 0.0
*B. longum* subsp. *longum*	Intestine of adult	ATCC 15707^T^	2.0 ± 0.4	1.1 ± 0.0
	Infant feces	ATCC BAA-999 (BB536)	4.1 ± 0.3	1.1 ± 0.1
infant-type HRB			3.2 ± 0.1	1.0 ± 0.0
*B. adolescentis*	Intestine of adult	ATCC 15703^T^	<0.005	1.2 ± 0.1
*B. angulatum*	Feces, human	ATCC 27535^T^	0.9 ± 0.3	1.0 ± 0.2
*B. dentium*	Dental caries	DSM 20436^T^	0.2 ± 0.1	1.0 ± 0.0
*B. pseudocatenulatum*	Feces, human	ATCC 27919^T^	0.2 ± 0.1	1.1 ± 0.0
adult-type HRB			0.4 ± 0.1 **	1.1 ± 0.0
*B. animalis* subsp. *lactis*	Yoghurt	DSM 10140^T^	0.2 ± 0.0	0.9 ± 0.0
*B. animalis* subsp. *animalis*	Rat feces	ATCC 25527^T^	0.2 ± 0.0	0.9 ± 0.0
*B. pseudolongum* subsp. *globosum*	Rumen, bovine	JCM 5820^T^	0.2 ± 0.1	0.7 ± 0.1
*B. pseudolongum* subsp. *pseudolongum*	Swine feces	ATCC 25526^T^	0.4 ± 0.0	0.8 ± 0.0
*B. thermophilum*	Swine feces	ATCC 25525^T^	0.6 ± 0.1	1.1 ± 0.1
non-HRB			0.3 ± 0.1 ^##^	0.9 ± 0.0

** Statistically significant difference in ILA production between infant-type HRB and adult-type HRB. ^##^ Statistically significant difference in ILA production between infant-type HRB and non-HRB. The rate of growth (OD600) and concentration of ILA in culture supernatants is shown. Values are expressed as means ± S.D.

**Table 2:** Production of ILA by 100 Human-Residential Bifidobacteria (HRB) Strains.

Strain	Total Number of Strains	ILA (µg/mL) in Culture Supernatants		
		Mean ± S.D. (µg/mL)	Range	
			Maximum	Minimum
*B. longum* subsp. *longum*	40	1.87 ± 1.05	4.92	0.05
*B. breve*	12	2.04 ± 0.97	3.85	0.46
*B. bifidum*	1	2.54	2.54	2.54
*B. kashiwanohense*	4	0.76 ± 1.21	2.57	0.09
infant-type HRB	57	1.84 ± 1.07 **	4.92	0.05
*B. pseudocatenulatum*	29	0.17 ± 0.08	0.33	0.03
*B. adolescentis*	13	0.21 ± 0.58	2.13	<0.005
*B. dentium*	1	0.2	0.2	0.2
adult-type HRB	43	0.4 ± 0.1	2.13	<0.005

** Statistically significant difference in ILA production between infant-type HRB and adult-type HRB.

## Supplementary Materials

The following are available online at https://www.mdpi.com/2076-2607/7/9/340/s1, Figure S1: Production of tryptophan metabolites (IAld, IAA, and IPA) by 19 bifidobacterial strains, Figure S2: Production of ILA by 100 bifidobacterial strains., Table S1: List of tryptophan metabolites and the internal standard., Table S2: Production of ILA by 19 bifidobacterial strains.

## References

[1] Gao J., Xu K., Liu H., Liu G., Bai M., Peng C., Li T., Yin Y. (2018). Impact of the gut microbiota on intestinal immunity mediated by tryptophan metabolism. Front. Cell. Infect. Microbiol..

[2] Roager H.M., Licht T.R. (2018). Microbial tryptophan catabolites in health and disease. Nat. Commun..

[3] Krishnan S., Ding Y., Saedi N., Choi M., Sridharan G.V., Sherr D.H., Yarmush M.L., Alaniz R.C., Jayaraman A., Lee K. (2018). Gut microbiota-derived tryptophan metabolites modulate inflammatory response in hepatocytes and macrophages. Cell Rep..

[4] Hou Q., Ye L., Liu H., Huang L., Yang Q., Turner J.R., Yu Q. (2018). *Lactobacillus* accelerates ISCS regeneration to protect the integrity of intestinal mucosa through activation of stat3 signaling pathway induced by LPLs secretion of IL-22. Cell Death Differ..

[5] Venkatesh M., Mukherjee S., Wang H., Li H., Sun K., Benechet A.P., Qiu Z., Maher L., Redinbo M.R., Phillips R.S. (2014). Symbiotic bacterial metabolites regulate gastrointestinal barrier function via the xenobiotic sensor PXR and Toll-like receptor 4. Immunity.

[6] Morshedi D., Rezaei-Ghaleh N., Ebrahim-Habibi A., Ahmadian S., Nemat-Gorgani M. (2007). Inhibition of amyloid fibrillation of lysozyme by indole derivatives--possible mechanism of action. FEBS J..

[7] Aoki-Yoshida A., Ichida K., Aoki R., Kawasumi T., Suzuki C., Takayama Y. (2013). Prevention of UVB-induced production of the inflammatory mediator in human keratinocytes by lactic acid derivatives generated from aromatic amino acids. Biosci. Biotechnol. Biochem..

[8] Suzuki Y., Kosaka M., Shindo K., Kawasumi T., Kimoto-Nira H., Suzuki C. (2013). Identification of antioxidants produced by *Lactobacillus plantarum*. Biosci. Biotechnol. Biochem..

[9] Cervantes-Barragan L., Chai J.N., Tianero M.D., Di Luccia B., Ahern P.P., Merriman J., Cortez V.S., Caparon M.G., Donia M.S., Gilfillan S. (2017). Lactobacillus reuteri induces gut intraepithelial CD4+ CD8αα+ T cells. Science.

[10] Wilck N., Matus M.G., Kearney S.M., Olesen S.W., Forslund K., Bartolomaeus H., Haase S., Mahler A., Balogh A., Marko L. (2017). Salt-responsive gut commensal modulates Th17 axis and disease. Nature.

[11] Peters A., Krumbholz P., Jager E., Heintz-Buschart A., Cakir M.V., Rothemund S., Gaudl A., Ceglarek U., Schoneberg T., Staubert C. (2019). Metabolites of lactic acid bacteria present in fermented foods are highly potent agonists of human hydroxycarboxylic acid receptor 3. PLoS Genet..

[12] Aragozzini F., Ferrari A., Pacini N., Gualandris R. (1979). Indole-3-lactic acid as a tryptophan metabolite produced by *Bifidobacterium* spp.. Appl. Environ. Microbiol..

[13] Smith E.A., Macfarlane G.T. (1996). Enumeration of human colonic bacteria producing phenolic and indolic compounds: Effects of pH, carbohydrate availability and retention time on dissimilatory aromatic amino acid metabolism. J. Appl. Bacteriol..

[14] Turroni F., Peano C., Pass D.A., Foroni E., Severgnini M., Claesson M.J., Kerr C., Hourihane J., Murray D., Fuligni F. (2012). Diversity of bifidobacteria within the infant gut microbiota. PLoS ONE.

[15] Ishikawa E., Matsuki T., Kubota H., Makino H., Sakai T., Oishi K., Kushiro A., Fujimoto J., Watanabe K., Watanuki M. (2013). Ethnic diversity of gut microbiota: Species characterization of *Bacteroides fragilis* group and genus *Bifidobacterium* in healthy Belgian adults, and comparison with data from Japanese subjects. J. Biosci. Bioeng..

[16] Odamaki T., Horigome A., Sugahara H., Hashikura N., Minami J., Xiao J.Z., Abe F. (2015). Comparative genomics revealed genetic diversity and species/strain-level differences in carbohydrate metabolism of three probiotic bifidobacterial species. Int. J. Genom..

[17] O’Callaghan A., van Sinderen D. (2016). Bifidobacteria and their role as members of the human gut microbiota. Front. Microbiol..

[18] Duranti S., Milani C., Lugli G.A., Turroni F., Mancabelli L., Sanchez B., Ferrario C., Viappiani A., Mangifesta M., Mancino W. (2015). Insights from genomes of representatives of the human gut commensal *Bifidobacterium bifidum*. Environ. Microbiol..

[19] Kato K., Odamaki T., Mitsuyama E., Sugahara H., Xiao J.Z., Osawa R. (2017). Age-related changes in the composition of gut *Bifidobacterium* species. Curr. Microbiol..

[20] Odamaki T., Bottacini F., Mitsuyama E., Yoshida K., Kato K., Xiao J.Z., van Sinderen D. (2019). Impact of a bathing tradition on shared gut microbe among Japanese families. Sci. Rep..

[21] Lin R., Liu W., Piao M., Zhu H. (2017). A review of the relationship between the gut microbiota and amino acid metabolism. Amino Acids.

[22] Liu Y., Alookaran J.J., Rhoads J.M. (2018). Probiotics in autoimmune and inflammatory disorders. Nutrients.

[23] Mashiguchi K., Tanaka K., Sakai T., Sugawara S., Kawaide H., Natsume M., Hanada A., Yaeno T., Shirasu K., Yao H. (2011). The main auxin biosynthesis pathway in *Arabidopsis*. Proc. Natl. Acad. Sci. USA.

[24] Gilbert S., Xu J., Acosta K., Poulev A., Lebeis S., Lam E. (2018). Bacterial production of indole related compounds reveals their role in association between duckweeds and endophytes. Front. Chem..

[25] Wong C.B., Sugahara H., Odamaki T., Xiao J.Z. (2018). Different physiological properties of human-residential and non-human-residential bifidobacteria in human health. Benef. Microbes.

[26] Morita H., Nakano A., Onoda H., Toh H., Oshima K., Takami H., Murakami M., Fukuda S., Takizawa T., Kuwahara T. (2011). *Bifidobacterium kashiwanohense* sp. Nov., isolated from healthy infant faeces. Int. J. Syst. Evol. Microbiol..

[27] Nishizawa T., Aldrich C.C., Sherman D.H. (2005). Molecular analysis of the rebeccamycin L-amino acid oxidase from *Lechevalieria aerocolonigenes* ATCC 39243. J. Bacteriol..

[28] Dodd D., Spitzer M.H., Van Treuren W., Merrill B.D., Hryckowian A.J., Higginbottom S.K., Le A., Cowan T.M., Nolan G.P., Fischbach M.A. (2017). A gut bacterial pathway metabolizes aromatic amino acids into nine circulating metabolites. Nature.

[29] Matteoli G., Mazzini E., Iliev I.D., Mileti E., Fallarino F., Puccetti P., Chieppa M., Rescigno M. (2010). Gut CD103^+^ dendritic cells express indoleamine 2,3-dioxygenase which influences T regulatory/T effector cell balance and oral tolerance induction. Gut.

[30] Lanz T.V., Becker S., Mohapatra S.R., Opitz C.A., Wick W., Platten M. (2017). Suppression of Th1 differentiation by tryptophan supplementation in vivo. Amino Acids.

[31] Thevaranjan N., Puchta A., Schulz C., Naidoo A., Szamosi J.C., Verschoor C.P., Loukov D., Schenck L.P., Jury J., Foley K.P. (2017). Age-associated microbial dysbiosis promotes intestinal permeability, systemic inflammation, and macrophage dysfunction. Cell Host Microbe.

[32] Franceschi C., Garagnani P., Parini P., Giuliani C., Santoro A. (2018). Inflammaging: A new immune-metabolic viewpoint for age-related diseases. Nat. Rev. Endocrinol..

[33] Shigeno Y., Zhang H., Banno T., Usuda K., Nochi T., Inoue R., Watanabe G., Jin W., Benno Y., Nagaoka K. (2019). Gut microbiota development in mice is affected by hydrogen peroxide produced from amino acid metabolism during lactation. FASEB J..

[34] Dieuleveux V., Lemarinier S., Gueguen M. (1998). Antimicrobial spectrum and target site of D-3-phenyllactic acid. Int. J. Food Microbiol..

