# Supplementary material for: Production of Indole-3-Lactic Acid by Bifidobacterium Strains Isolated fromHuman Infants
Source: Microorganisms. 2019 Sep 11;7(9):340. doi: 10.3390/microorganisms7090340 (PMC6780619; doi:10.3390/microorganisms7090340)
Supplement: Supplementary file 1 [file microorganisms-07-00340-s001.zip › Supplementary Files/Figure S1.pptx]

## Slide 1
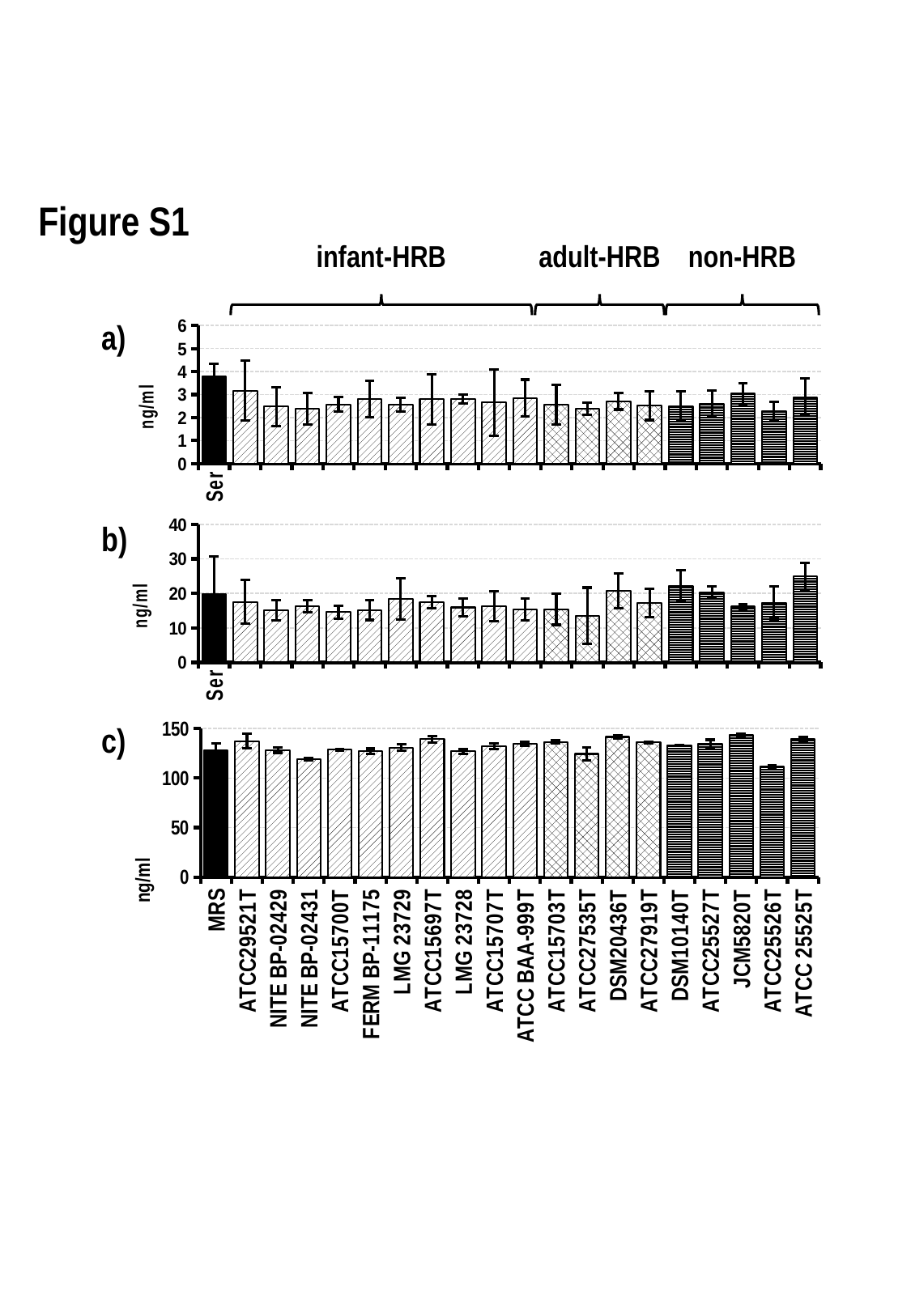

Figure S1
infant-HRB
adult-HRB
non-HRB
a)
### Chart
| Category | IPA |
|---|---|
| | 3.7808836053410935 |
| | 3.1737999913816144 |
| | 2.4737670580751328 |
| | 2.385213689328042 |
| | 2.573323833240045 |
| | 2.807541800612586 |
| | 2.5599306925364793 |
| | 2.79282395930021 |
| | 2.810455086453873 |
| | 2.659102555974867 |
| | 2.8522930617458853 |
| | 2.561298600110695 |
| | 2.383930675411282 |
| | 2.7145412429260056 |
| | 2.523626933393281 |
| | 2.4790529482989907 |
| | 2.5972748943435944 |
| | 3.0407426528683636 |
| | 2.268872536904405 |
| | 2.884629722348719 |
### Chart
| Category | IAA |
|---|---|
| | 19.874400380898976 |
| | 17.49859508641576 |
| | 15.066837929994467 |
| | 16.347873334872347 |
| | 14.541134558365131 |
| | 15.184864464100759 |
| | 18.361240698144282 |
| | 17.45053074319387 |
| | 15.914515817912838 |
| | 16.264861379669046 |
| | 15.322840143035148 |
| | 15.388676623110513 |
| | 13.472878379589346 |
| | 20.670696435744603 |
| | 17.21664146379448 |
| | 22.17325090911757 |
| | 20.29573590521093 |
| | 16.156672908774052 |
| | 17.266494369213085 |
| | 24.85526017164359 |b)
### Chart
| Category | IAld |
|---|---|
| MRS | 127.9316186926306 |
| ATCC29521T | 137.42292999541178 |
| NITE BP-02429 | 128.2604286071488 |
| NITE BP-02431 | 119.1573984199555 |
| ATCC15700T | 128.61114056745947 |
| FERM BP-11175 | 127.1128665269066 |
| LMG 23729 | 130.69141700479292 |
| ATCC15697T | 139.229368230966 |
| LMG 23728 | 127.02716283424806 |
| ATCC15707T | 132.41966905794885 |
| ATCC BAA-999T | 134.97423185772527 |
| ATCC15703T | 136.53263045342197 |
| ATCC27535T | 124.48437992308075 |
| DSM20436T | 141.62991701737747 |
| ATCC27919T | 136.11019241533995 |
| DSM10140T | 133.0749938351039 |
| ATCC25527T | 134.44458744677053 |
| JCM5820T | 143.64943013927655 |
| ATCC25526T | 111.5133283375418 |
| ATCC 25525T | 139.79181500896817 |c)
