# Supplementary material for: Production of Indole-3-Lactic Acid by Bifidobacterium Strains Isolated fromHuman Infants
Source: Microorganisms. 2019 Sep 11;7(9):340. doi: 10.3390/microorganisms7090340 (PMC6780619; doi:10.3390/microorganisms7090340)
Supplement: Supplementary file 1 [file microorganisms-07-00340-s001.zip › Supplementary Files/Figure S2.pptx]

## Slide 1
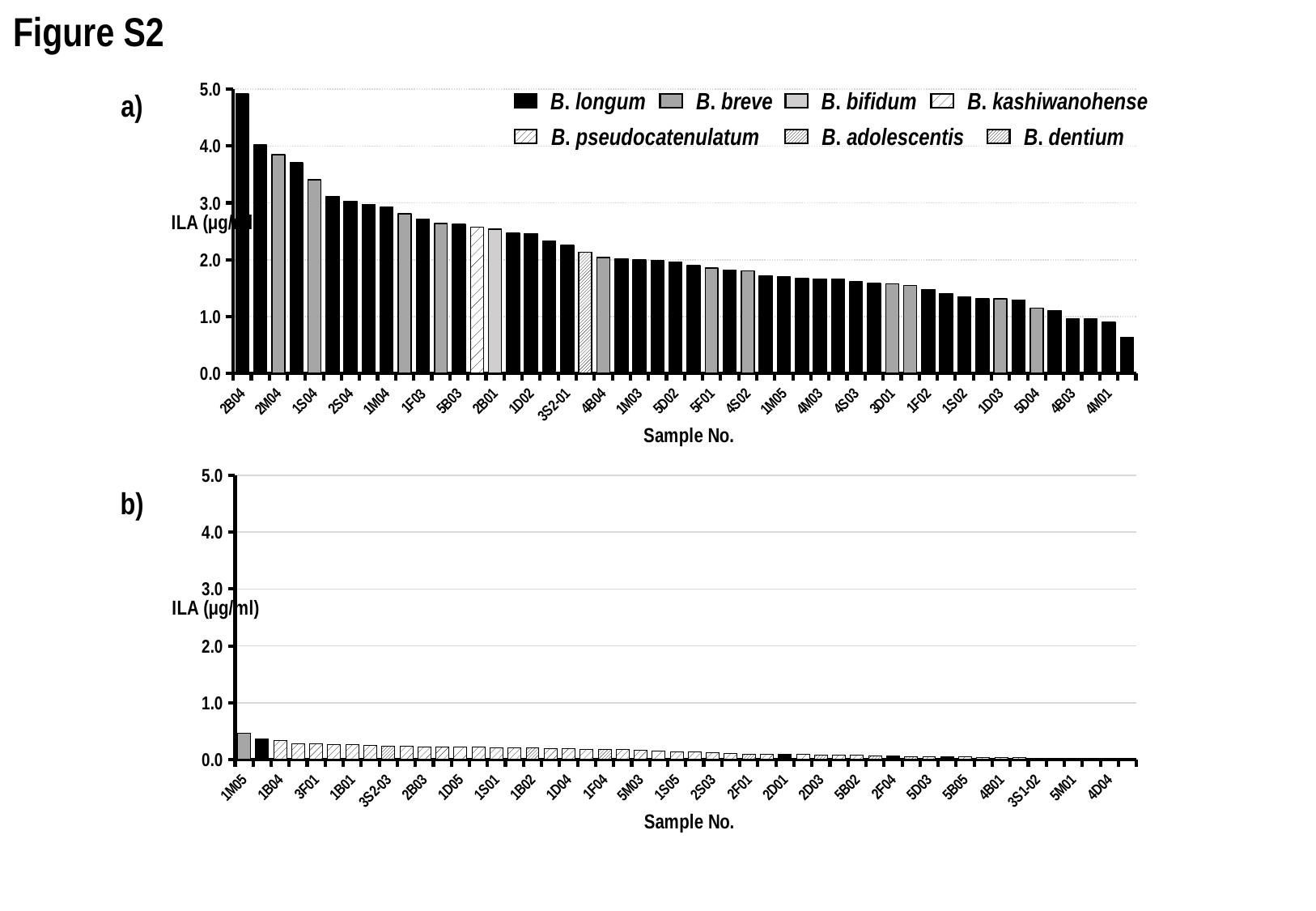

Figure S2
### Chart
| Category | |
|---|---|
| 2B04 | 4.917007987998458 |
| 4S01 | 4.017629999816372 |
| 2M04 | 3.8462443837057987 |
| 3B02 | 3.708714489765878 |
| 1S04 | 3.4050033262935657 |
| 2D02 | 3.1135957020071063 |
| 2S04 | 3.027037181642822 |
| 2M02 | 2.972880189815824 |
| 1M04 | 2.9187988267562837 |
| 3B01 | 2.8077454897796774 |
| 1F03 | 2.717330442090283 |
| 5B01 | 2.6366081957196754 |
| 5B03 | 2.620238914505749 |
| 1M01 | 2.5739845337211276 |
| 2B01 | 2.537256827914683 |
| 5D05 | 2.464081941328401 |
| 1D02 | 2.462277541276078 |
| 3F02 | 2.3301836317449807 |
| 3S2-01 | 2.2522787850293424 |
| 3F03 | 2.13269137533422 |
| 4B04 | 2.038501818505705 |
| 5F03 | 2.015174040890123 |
| 1M03 | 1.9995573564998352 |
| 4F02 | 1.9787916311372276 |
| 5D02 | 1.9608721185419784 |
| 1B03 | 1.8993459754253705 |
| 5F01 | 1.8554536333346314 |
| 5M02 | 1.8142654636877145 |
| 4S02 | 1.8062174740851187 |
| 4B02 | 1.720113582237814 |
| 1M05 | 1.6998365427206732 |
| 1D01 | 1.671892935762568 |
| 4M03 | 1.661273461674818 |
| 5S01 | 1.6554766284700215 |
| 4S03 | 1.6139377442116125 |
| 4D02 | 1.5816037572035622 |
| 3D01 | 1.5723247226576802 |
| 5S02 | 1.5461820901742072 |
| 1F02 | 1.473204909181225 |
| 2B02 | 1.4036604986590633 |
| 1S02 | 1.3425673040965604 |
| 3M02 | 1.3152333866302843 |
| 1D03 | 1.311699884514105 |
| 3S2-02 | 1.29016924605016 |
| 5D04 | 1.1451158490534838 |
| 5F02 | 1.105550992642753 |
| 4B03 | 0.9643161098921844 |
| 4D03 | 0.9565954206501077 |
| 4M01 | 0.8960140477788751 |
| 5B04 | 0.6373309011881719 |a)
B. longum
B. breve
B. bifidum
B. kashiwanohense
B. pseudocatenulatum
B. adolescentis
B. dentium
### Chart
| Category | |
|---|---|
| 1M05 | 0.46196528760170064 |
| 2F02 | 0.36671392974121225 |
| 1B04 | 0.3334607747805185 |
| 1F01 | 0.27339880383471166 |
| 3F01 | 0.2711456743014592 |
| 3M01 | 0.26940026061203554 |
| 1B01 | 0.25752321901786585 |
| 1M02 | 0.24837350230039207 |
| 3S2-03 | 0.232713227917385 |
| 2M01 | 0.23045306666349738 |
| 2B03 | 0.22535642400120937 |
| 1D06 | 0.22061253628800925 |
| 1D05 | 0.2159404953195843 |
| 2F03 | 0.21350020405279008 |
| 1S01 | 0.21265664267087686 |
| 2M03 | 0.2067718697808037 |
| 1B02 | 0.2036798957058935 |
| 1D07 | 0.1950809354556028 |
| 1D04 | 0.1868802965573614 |
| 2S01 | 0.17585754927718492 |
| 1F04 | 0.17347949028241094 |
| 1M06 | 0.17316767442244932 |
| 5M03 | 0.16120579792279638 |
| 2S02 | 0.14783297867219353 |
| 1S05 | 0.13944317429847192 |
| 5F04 | 0.12814938029030898 |
| 2S03 | 0.12661187432287213 |
| 4F01 | 0.10328921741483989 |
| 2F01 | 0.09665838602490032 |
| 4M02 | 0.09526927665175225 |
| 2D01 | 0.08904315328686424 |
| 1S03 | 0.08755659051706499 |
| 2D03 | 0.08346107976822446 |
| 4F04 | 0.07766713438130975 |
| 5B02 | 0.07459156378365502 |
| 3M03 | 0.06621951863078905 |
| 2F04 | 0.05996195415292691 |
| 3F04 | 0.05490506977122943 |
| 5D03 | 0.053059099179794865 |
| 3F05 | 0.04565385619827213 |
| 5B05 | 0.043217108123628716 |
| 4S04 | 0.041786877012122914 |
| 4B01 | 0.03523298902766918 |
| 4D01 | 0.03233891917774481 |
| 3S1-02 | 0.02157339951236 |
| 5D01 | 0.0 |
| 5M01 | 0.0 |
| 3S1-01 | -0.0080111020138591 |
| 4D04 | -0.0489064704050755 |
| 4F03 | -0.0779537248197444 |b)
